# Glial-Plug Proliferation after Inverted Internal Limiting Membrane Flap Technique for Idiopathic Macular Hole

**DOI:** 10.1155/2022/2919358

**Published:** 2022-09-30

**Authors:** Sławomir Cisiecki, Karolina Bonińska, Tomasz Dybek, Maciej Bednarski

**Affiliations:** ^1^Centrum Medyczne, Julianów Ul Żeglarska 4, 91-321 Łodz, Poland; ^2^Miejskie Centrum Medyczne, Ul Milionowa 14, 93-113 Łodz, Poland

## Abstract

**Purpose:**

This study aimed to analyze the effect of multiple folded internal limiting membrane (ILM) flap in the inverted ILM flap technique on postoperative outcomes of patients with full-thickness macular hole (FTMH).

**Methods:**

This retrospective study included 41 eyes of 41 patients with FTMH treated with vitrectomy using the inverted ILM flap technique. Complete ophthalmic examination was performed preoperatively, at 1 week, and at 1, 3, 6, 9, and 12 months after surgery.

**Results:**

Postoperative SD-OCT confirmed macular hole (MH) closure in all patients. The mean BCVA improved from 0.7 LogMAR (20/100) preoperatively to 0.5 LogMAR (20/63) postoperatively. In two cases, 7 days after vitrectomy, flap closure was noted. At the final visit after 12 months, the following foveal contours were noted: 14 U-shape (34.1%), 12 irregular (W-shape) (29.3%), and 6 V-shape (14.6%). We observed a type of “plug closure” in 9 (22%) eyes in which hyperproliferation was noted in one eye.

**Conclusions:**

The surgeons should be aware of potential hyperproliferation on the retinal surface after the multi-layered flap technique.

## 1. Introduction

Although vitrectomy with inner limiting membrane (ILM) peeling remains the gold standard in the case of full-thickness macular hole (FTMH), the technique of inverted ILM flap is playing an increasingly important role. Some surgeons reserve it for the most demanding cases, such as large holes (>400 *μ*m) and persistent or high myopia while others use it in all FTMH cases [1–7].

This technique was first described in 2009 and has since gone through many modifications [8].

The ILM flap technique has many variations. There are temporal ILM flap in which the ILM is peeled 1 off only from the temporal side of the fovea, the cabbage leaf ILM flap technique in which multiple ILM flaps are inverted over each other and the hole like cabbage leaves, or free flap harvested from the surrounded retina [1, 9, 10]. In the primary classical version of this technique, the ILM is peeled for approximately two disc diameters and the remaining is massaged over the MH from all sides until it becomes inverted [1]. Instead of a single-layered flap, it is a multi-layered ILM folded flap technique.

The exact mechanisms of macular hole closure and retinal restoration remain unknown [11–13].

Therefore, we investigated whether multiple folded ILM in the classical inverted ILM flap technique has an effect on postoperative anatomical and functional outcomes in patients with FTMH.

## 2. Materials and Methods

We retrospectively reviewed the medical records of all patients who underwent surgery for FTMH repair using the inverted ILM flap technique at Julianow Medical Center between 2016 and 2020. All patients were operated on by one surgeon (SC). Only patients with idiopathic FTMH in stage 4 according to Gass were included. All our patients were pseudophakic. Finally, 41 eyes of 41 patients (33 females (80.5%) and 8 males (19.6%) with a mean age of 65.9) were included. All patients signed an informed consent form prior to the surgery. The study was in accordance with the Declarations of Helsinki and the institutional guidelines.

All patients underwent a comprehensive ophthalmologic evaluation, including measurement of best-corrected visual acuity (BCVA) using Early Treatment Diabetic Retinopathy Study charts, intraocular pressure measurements, slit-lamp biomicroscopy, and SD-OCT (HRA + OCT Spectralis System, Heidelberg Engineering, Germany), at baseline, at 7 days, and at least 1, 3, 6, 9, and 12 months postoperatively.

Pre and postoperative SD-OCT measurements were performed. The minimum linear and base diameters of the MH and the foveal configurations were assessed using SD-OCT. The minimum linear diameter (minimal extent of the MH parallel to the RPE) and the maximum base diameter (diameter at the level of the RPE) were measured.

### 2.1. Surgical Procedure

A standard three-port 23-gauge pars plana vitrectomy with an inverted ILM flap technique with periocular anesthesia was performed in all patients. After core vitrectomy, the ILM was stained with Membrane Dual Blue® (Dorc, Rotterdam) for 20 s directly under fluid without air-fluid exchange. In a few cases during staining, there were signs of some “negative staining.” Thereafter, the ILM was grasped from all four sites of the hole and peeled off until the margin of the hole using ILM forceps, leaving the innermost part attached at the rim of the MH.

Subsequently, it was stabilized using a perfluorocarbon liquid (PFCL) bubble. During this maneuver, perfusion was set at a low level. The remnants of the ILM were reinverted to cover the MH under the PFCL bubble. After checking the peripheral retina, fluid-air exchange was performed with meticulous aspiration of the remaining BSS around the PFCL bubble. At the end of surgery, the PFCL bubble was aspirated and patients were asked to maintain a face-down position for 1 day postoperatively.

Our surgical technique differed slightly from that of the original version. First, if an epiretinal membrane was present (which was only suspected by the negative staining phenomena intraoperatively), it was not removed separately because of the risk of the combined peeling with ILM below which could result in the loss of ILM. Second, peripheral remnants of the ILM were not trimmed with a vitreous cutter.

### 2.2. Statistical Analysis

The statistical analysis encompassed generalized linear models with robust standard errors and the Kruskal–Wallis test. Two-sided procedures were performed. Statistical significance was set at *P* < 0.05. Statistical analyses were performed using Stata/Special Edition, release 14.2 (StataCorp LP, College Station, Texas, USA).

## 3. Results

**Figure 1:**
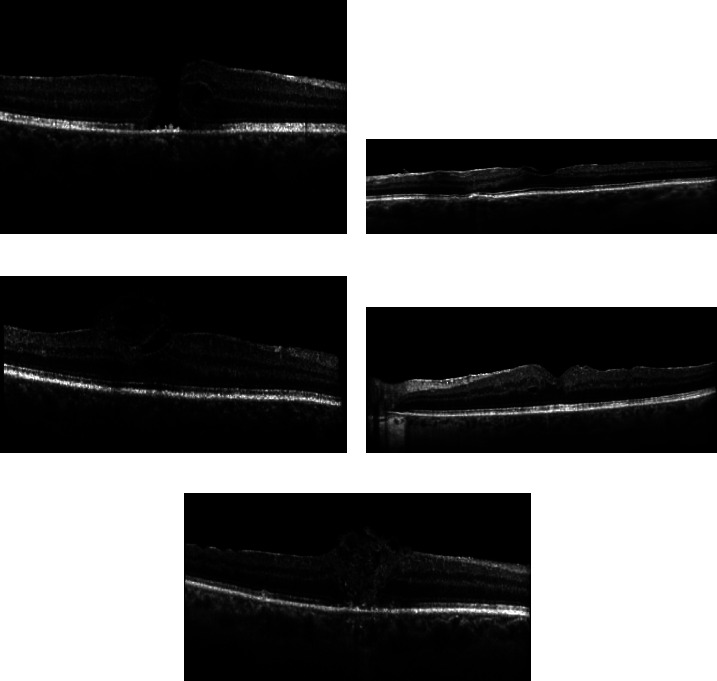
SD-OCT image demonstrates the foveal contour classified as (a) flap closure, (b) U-shape, (c) W-shape, (d) V-shape, and (e) plug closure at the final visit.

Postoperative SD-OCT scans confirmed MH closure in all cases. No flat-open closure was observed. In two cases, 7 days after vitrectomy, flap closure was observed (Figure 1(a)). No adverse events were recorded during the surgery or a follow-up period of 12 months.

On SD-OCT examination at baseline, the mean minimal MH diameter was 466.8 *μ*m, and the mean MH base diameter was 947.5 *μ*m. There was no evidence of ERM formation preoperatively.

The mean BCVA improved from 0.7 LogMAR (Snellen's equivalent 20/100) preoperatively to 0.5 LogMAR (Snellen's equivalent 20/63) postoperatively.

**Figure 2:**
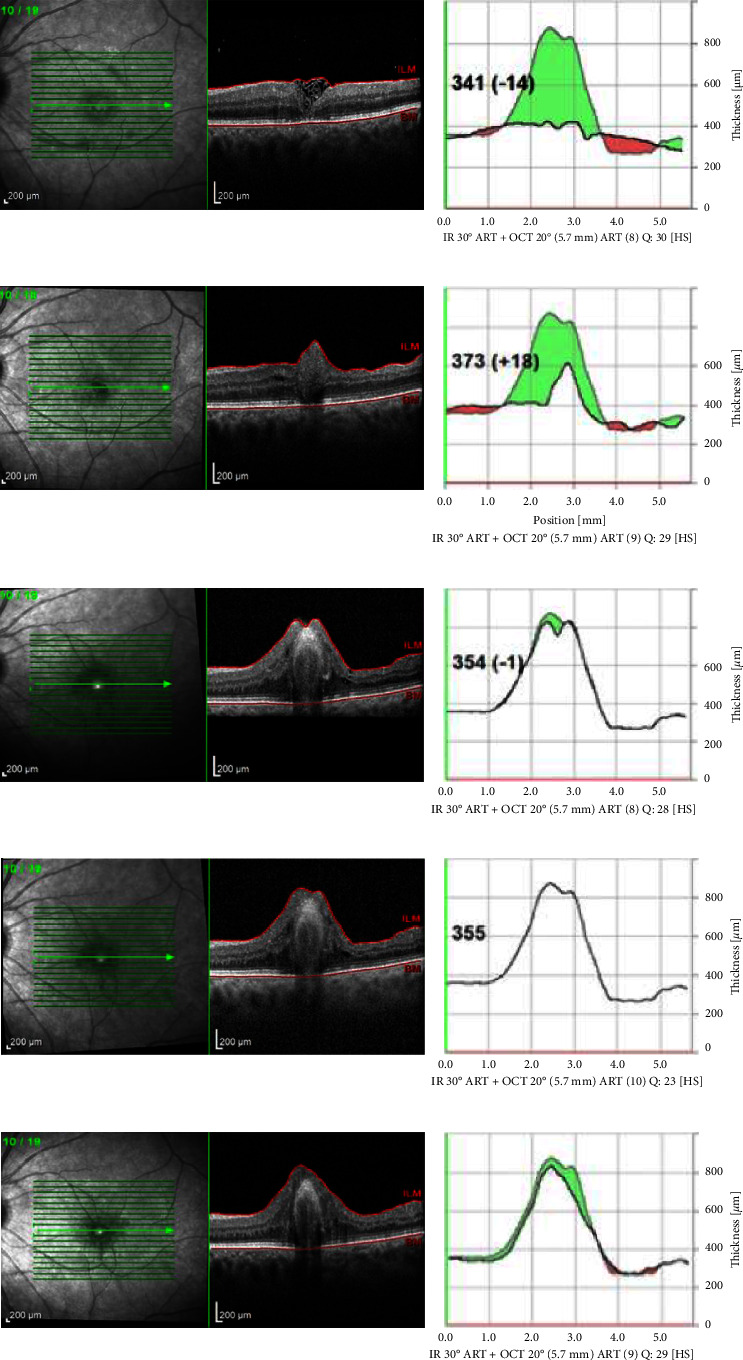
Foveal architecture changes in a patient with hyperproliferation. SD-OCT: (a) 1 month postoperatively; (b) 3 months postoperatively; (c) 6 months postoperatively; (d) 9 months postoperatively; (e) 1 year postoperatively.

Changes in the foveal contour were also analyzed. At the final visit after 12 months, the following foveal contours were noted: 14 U-shape (34.1%) (Figure 1(b)), 12 irregular (W-shape) (29.3%) (Figure 1(c)), and 6 V-shape (14.6%) (Figure 1(d)). In 9 (22%) eyes, we observed a type of plug closure (Figure 1(e)). In this group, hyperproliferation was noted in one eye (Figures 2(a)–2(d)).

**Figure 3:**
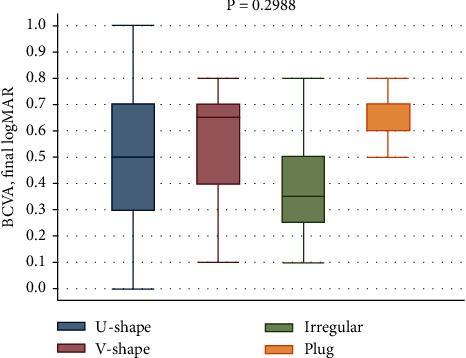
Correlation between each foveal contour and final BCVA.

We found an improvement in the BCVA during the follow-up period in each foveal contour (*P*=0.2988) (Figure 3). The worst final BCVA was noted in the plug closure group.

**Figure 4:**
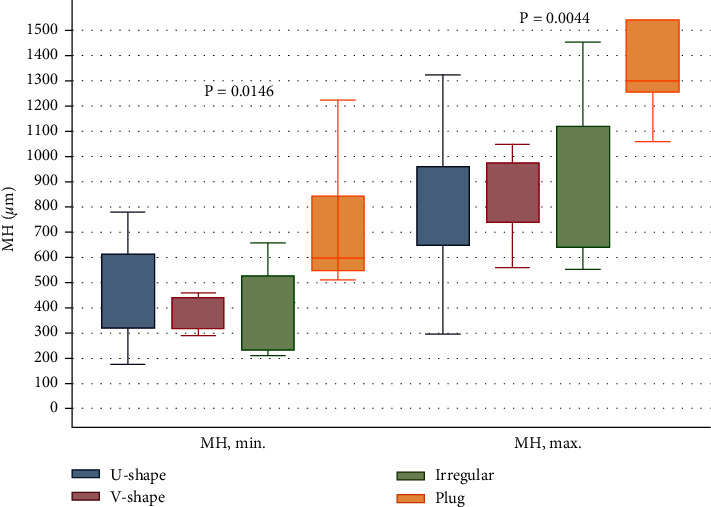
Correlation between minimal and base diameters and foveal contour at the final visit.

The correlation between average final BCVA and minimal diameter (*P*=0.2128) as well as base diameter (*P*=0.6716) was not significant. Plug closure was observed in the macular holes with large preoperative diameters (Figure 4).

## 4. Discussion

In histopathological terms, macular hole closure using the inverted ILM flap technique is multi-factorial. The ILM contains Müller cells that, through neurotrophic factors and basic fibroblast growth factor, induce gliosis that facilitates the closure of the hole [14]. In addition, the ILM flap acts as a scaffold to the surrounding tissues, facilitating the proliferation and migration of Müller cells and closure of the hole [14].

Our study suggests that this gliosis may not be limited to the MH only. The multi-layered flap technique may increase the risk of this hyperproliferation on the retinal surface.

Similar observations were reported by Shiode et al. They reported that gliosis by Müller cells is effective in closing MHs; however, excessive gliosis has cytotoxic effects on retinal neurons and may indicate a worse visual prognosis. Although foveal hyperreflective lesions usually disappear within a few months postoperatively, persistent activation of glial cells may contribute to tissue damage and result in scar formation [14].

Kanda et al. reported two cases of macular proliferation after inverted ILM flap technique [15]. Histopathological examination of the surgical specimens indicated strong proliferation between the ILMs inside the flap. They find it is necessary to consider that the tissues that form on the ILM may lead to macular pucker formation following this technique [15].

In our study, in 22% of cases (9 eyes), we observed such a scar in the fovea called the “plug closure” (Figure 1(e)). These lesions persisted during the follow-up period and were associated with worse visual acuity. Moreover, in this group, hyperproliferation was noted in one eye (Figures 2(a)–2(d)). We suspect that this hyperproliferation may not only be due to increased Müller cell activity but also due to the co-occurrence of the epiretinal membrane self-formation which can multiply if we used more layers of the ILM flap.

In all cases, there was no ERM formation in SD-OCT preoperatively; however, during staining, there were signs of some “negative staining.” It might indicate a proliferation island on the surface of ILM, which might grow later and cause ERM formation after performing ILM/ERM flaps. Removal of ERM separately before ILM peeling is very often impossible because of the risk that both structures will be peeled together, and therefore, there is not sufficient ILM flap to cover the hole. According to our experience, we treated many cases with ILM/ERM flaps together, and in the majority of cases, we did not observe such a phenomenon.

A single-layer flap seems to be the safest solution to avoid plug proliferation. However, this is associated with the highest risk of flap displacement through the remaining fluid in the vitreous cavity.

Our study contains some limitations, like the lack of a control group and small size group, and we did not analyze other potential ocular risk factors of hyperproliferation; however, this is a very rare complication, and hence, it is not easy to compare it with a control group. Further studies are required to identify the risk factors for gliosis and develop a method to prevent these excessive processes.

In conclusion, recent developments in surgical techniques in macular hole surgery, such as the multiple folded ILM in the classical inverted ILM flap technique, not only increase the chances of anatomical and functional success but also allow the observation of new retinal repair mechanisms.

The surgeons should be aware of potential hyperproliferation in cases where any epiretinal membrane formation is detected, only suspected by the negative staining phenomena intraoperatively. In those cases, classical ILM removal with subsequent gas tamponade or single-layer inverted flap technique should be considered.

## Data Availability

No data were used to support this study.

## Additional Points

The inverted internal limiting membrane flap technique improves the anatomical and functional results of full-thickness macular holes. We observed “plug closure” in nine eyes of 41 patients treated with this technique in which one eye showed hyperproliferation. Our study suggests that gliosis is not limited to the macular hole only.
